# Engaging with peers to integrate community care: Knowledge synthesis and conceptual map

**DOI:** 10.1111/hex.14034

**Published:** 2024-04-03

**Authors:** Andreea‐Cătălina Panaite, Odile‐Anne Desroches, Émilie Warren, Ghislaine Rouly, Geneviève Castonguay, Antoine Boivin

**Affiliations:** ^1^ Canada Research Chair in Partnership with Patients and Communities CHUM Research Center Montréal Québec Canada; ^2^ School of Public Health Université de Montréal Montréal Québec Canada; ^3^ Department of Family Medicine Université de Montréal Montréal Québec Canada

**Keywords:** community health, community health worker, integrated care, patient engagement, patient navigator, peers, peer support

## Abstract

**Context:**

Engaging with peers is gaining increasing interest from healthcare systems in numerous countries. Peers are people who offer support by drawing on lived experiences of significant challenges or ‘insider’ knowledge of communities. Growing evidence suggests that peers can serve as a bridge between underserved communities and care providers across sectors, through their ability to build trust and relationships. Peer support is thus seen as an innovative way to address core issues of formal healthcare, particularly fragmentation of care and health inequalities. The wide body of approaches, goals and models of peer support speaks volumes of such interest. Navigating the various labels used to name peers, however, can be daunting. Similar terms often hide critical differences.

**Objectives/Background:**

This article seeks to disentangle the conceptual multiplicity of peer support, presenting a conceptual map based on a 3‐year knowledge synthesis project involving peers and programme stakeholders in Canada, and international scientific and grey literature.

**Synthesis/Main Results:**

The map introduces six key questions to navigate and situate peer support approaches according to peers' roles, pathways and settings of practice, regardless of the terms used to label them. As a tool, it offers a broad overview of the different ways peers contribute to integrating health and community care.

**Discussion:**

We conclude by discussing the map's potential and limitations to establish a common language and bridge models, in support of knowledge exchange among practitioners, policymakers and researchers.

**Patient or Public Contribution:**

Our team includes one experienced peer support worker. She contributed to the design of the conceptual map and the production of the manuscript. More than 10 peers working across Canada were also involved during research meetings to validate and refine the conceptual map.

## 1 INTRODUCTION

People with lived experiences have engaged in care for hundreds of years, internationally. Self‐help groups for people experiencing mental illness, addictions or poverty are documented in the seventeenth and eighteenth centuries in France, Britain and the United States.^1^, ^2^, ^3^, ^4^ In the first half of the twentieth century, community members also mobilized in Asian and South East Asian countries to educate on health and improve access to care.^5^, ^6^ In the late 1960s and 1970s, peer programmes were launched to reach drug‐user communities in the United States and Western Europe.^7^ More broadly, mutual aid among community members and people sharing identities (e.g., women, youth, the elderly) was fundamental to the survival of civilizations.^2^, ^8^

Peer support is not a new idea or practice, particularly in informal community settings. It is, however, increasingly implemented in formal healthcare systems, gaining attention from providers, health representatives and policymakers in different countries.^2^, ^9^ Research suggests that peers can complement professional healthcare and address some of its shortcomings, notably inequities in care and fragmentation of services.^10^, ^11^ Peer support programmes have been launched in numerous settings, for instance, clinics, hospitals, schools, community and peer‐run organizations, to reach a variety of communities, such as people experiencing homelessness, chronic illnesses, migrants and refugees, or Indigenous people.^12^, ^13^, ^14^, ^15^ Engaging with peers is thus seen as an innovative way to integrate health and community care, cutting across contexts, settings and populations.

In healthcare ecosystems, peers hold a unique position. They mobilize lived experiences of hardships, life challenges or ‘insider’ knowledge of communities to support others.^2^, ^15^ Growing evidence suggests that through their ability to build trust and relationships, peers can serve as a bridge between communities and formal healthcare, particularly for those marginalized and disadvantaged.^2^, ^11^, ^16^ Peers are able to coordinate services between health and social care sectors and help with follow‐ups or referrals to providers or organizations.^17^ Research suggests that they also contribute to the self‐management and coping skills of individuals facing challenges,^15^, ^18^ empowering them to achieve their goals.^2^ Peers can advocate for the actual needs of underserved communities, and sensitize health professionals and representatives to the barriers faced in care.^19^, ^20^ Engaging with peers in care aligns with the increased recognition that community participation and social relationships are pillars of health promotion.^21^

The number of international and local initiatives speaks volumes to the interest in, and value of, peer support. In 2014, the World Health Organization reported the existence of self‐help groups across the globe, notably for substance use (Alcoholics Anonymous, Narcotics Anonymous, Cocaine Anonymous) and family support groups.^22^ There were approximately 700 programmes engaging peers in England alone in the early 2000s,^12^ and up to one million community health workers in China in 2010.^23^

**Figure 1:**
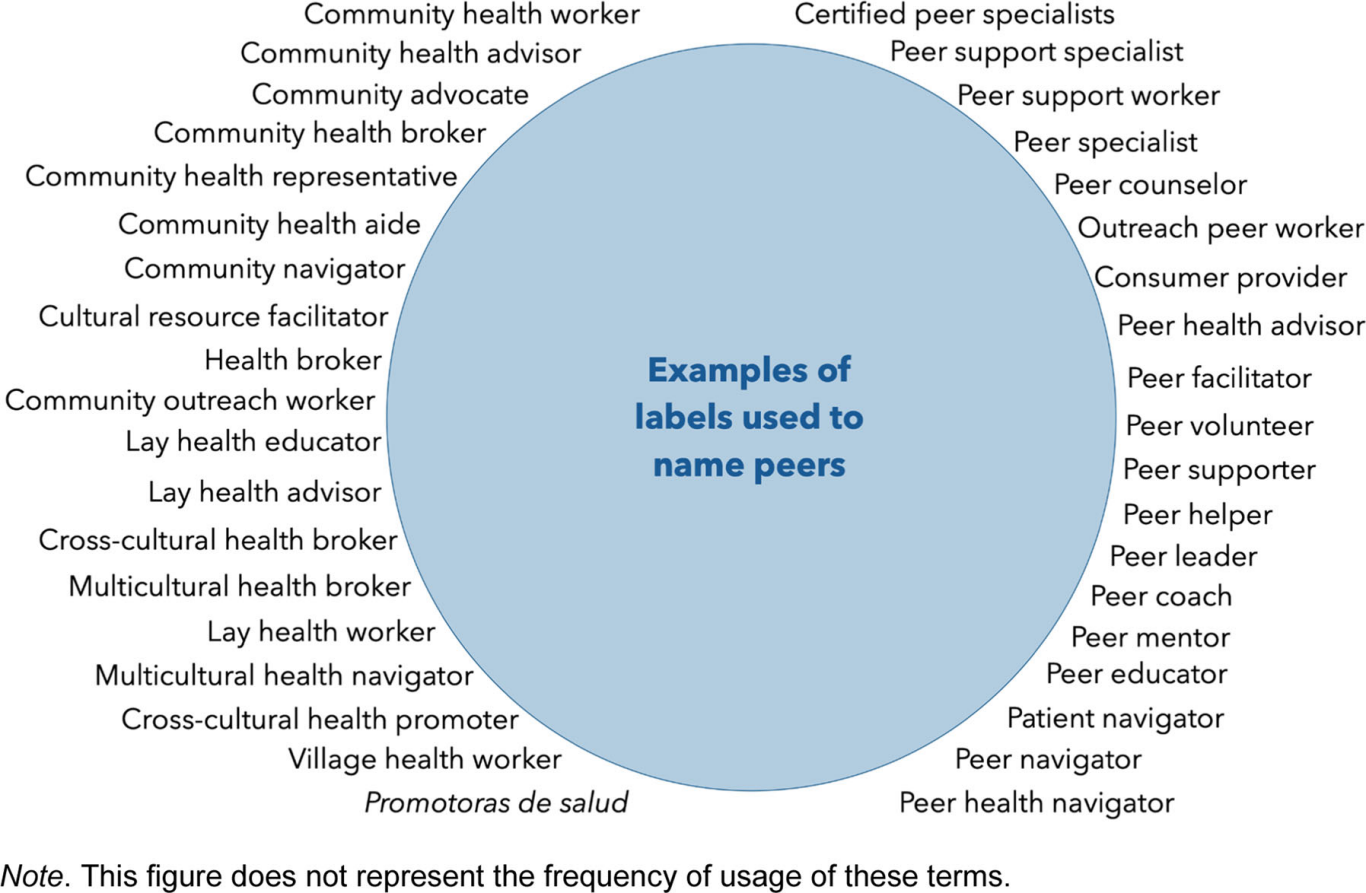
Multiplicity of terms used to describe peers.

Navigating the abundance of initiatives can be daunting at first. Peer roles are spread into a heterogeneous body of approaches, goals and models. There seems to be no shortage of names to describe peers: peer support workers, peer counsellors, peer mentors, peer navigators, lay health educators, community health workers, outreach workers, peer health advisors, health brokers and so on (see Figure 1). This diversity prompts different questions. Do peer mentors have the same background as community health workers? What makes them peers? Do they work in similar settings? Why should someone recruit one rather than the other? Finding answers to these questions requires processing multiple notions and concepts. This entails different implications for programme stakeholders, researchers and peers.

For policymakers or health system managers, this diversity makes it difficult to pinpoint which model is best suited to reach a target group or grasp the possibilities to explore at the outset. For researchers and academics, it challenges extensive investigation of the subject across approaches and constrains overviews of the implementation and effectiveness of peer interventions. Finally, for peers, it can limit their ability to recognize each other and learn from one another, as they may hold similar functions but have different work titles.

Albeit confusing, the variety of terms used to name peers is also a testimony to their versatile role in community health and to the rich history of peer support. To help navigate this multiplicity, we developed a conceptual map based on a knowledge synthesis. The project involved peers, practitioners and researchers in the field, and exploration of international scientific and grey literature. The purpose of the conceptual map is to provide a tool to approach the multifaceted forms of peers' engagement in care and bridge the diversity of models.

This paper is structured around three sections. First, we provide information on the methods of knowledge synthesis. Second, we present the conceptual map, starting with a brief detour through historical trends of peer support to contextualize this tool. Third, we conclude by discussing the potential and limitations of the map to offer a common language and facilitate knowledge exchange between practitioners, policymakers and researchers, regardless of the terms or labels used. Throughout the paper, we choose to use the term ‘peer’, and define it broadly to include different practices where individuals offer support based on lived experiences or ‘insider’ knowledge of communities.^2^, ^15^

## 2 METHODS

To develop the conceptual map, we conducted a 3‐year knowledge synthesis (2021–2023). Our approach was inspired by three research traditions: (1) narrative reviews, which emphasize the use of a comprehensive approach to broad topics^24^; (2) enrichment and contextualization of research data through the use of deliberative processes with peers, practitioners and researchers^25^; (3) participatory research approaches, which aim to produce knowledge based on continuous and iterative collaboration between researchers, practitioners and communities.^26^

Concretely, our knowledge synthesis consisted of two iterative steps: exploring international scientific and grey literature and building the conceptual map with peers, practitioners and researchers involved in peer support in Canada. Because we sought to bridge different literature on peers' engagement in care, using a systematic or narrow search strategy with strictly predefined terms and questions did not seem appropriate.^24^ We thus opted for a purposeful, iterative and flexible approach to literature and knowledge synthesis, as it provided the comprehensive and narrative posture this project required.^24^, ^27^

The first step consisted of exploring the scientific and grey literature (2021). We ran the first set of inquiries in two bibliographic databases (PubMed and Google Scholar) using three keywords (peer support worker, community health worker, patient navigator). We decided to mainly include literature reviews or overview articles with a focus on primary care and/or integrated care. Reports (grey literature) were identified through organizational websites (e.g., World Health Organization). We supplemented our results by ‘snowballing the search’ (i.e., screening references in and of included papers). Articles and reports were selected (*n* = 81) based on their usefulness to capture multiple facets of peers' engagement in care across contexts and traditions. After screening the content of selected documents, we removed 13 articles and seven reports, as they did not provide sufficient information to capture diverse dimensions of peer interventions (e.g., focused on feasibility or effectiveness of a narrow task, offered practical guidelines or studied healthcare providers offering navigational support).

The second step consisted of building the conceptual map. We conducted an in‐depth review of each selected document (58 articles and three reports), outlining and selecting descriptive themes of peers' interventions (roles, experiences, background, training, settings, purpose, programme history), while accounting for variations across traditions and models. We then identified an initial set of key questions to regroup these themes. Co‐authors, which include a peer with more than 50 years of experience in peer support in community care, met to discuss. After reaching consensus on the key questions that would form the conceptual map, we went back to our articles to (re)organize themes and content using the questions identified. Four iterations of the conceptual map (2022–2023) were subsequently discussed with co‐authors, going back and forth between redefining key questions and thematic reorganization of articles' content. Throughout this process, we removed articles (*n* = 33) that did not provide sufficient information to build on the evolving iterations of the conceptual map. In addition, we used PubMed for focused inquiries (on informal peer support and self‐help groups), and ‘snowballed the search’ to add articles (*n* = 25) that enabled us to build across iterations of the conceptual map. This led us to the final version of our conceptual map (including a corpus of 50 articles and three reports).

During the second step, the team sought to understand how the map related (or not) to other peers currently engaged in care. We met twice with 12 practising peers and two healthcare professionals working with different communities across Canada (Indigenous health, harm reduction, mental health, the health of people at risk or experiencing homelessness, migrant health, cancer, ageing, end of life and primary care). We presented the conceptual map and asked participants to comment on its relevance based on their experiences. Peers reflected that the map provided an overview of important themes, but encouraged us to highlight how peers' engagement was a direct response to shortcomings of formal healthcare systems, particularly with marginalized communities (e.g., mistrust, health inequalities or racism). We adjusted the work accordingly and met again to ensure we understood their comments and integrated them appropriately.

## 3 HISTORICAL TRENDS

Peers' history and contribution to community health predates the development of formal health professional roles. For this article, we focused on three contemporary trends of peers' engagement in care: peer support, community health workers and patient navigation programmes. Each highlight how social, historical, cultural and geopolitical contexts shape the way health ecosystems have partnered with peers in care.

Peer support, or support by people with lived experiences of significant life challenges, has been traced back to mutual aid societies and grassroots advocacy groups for marginalized communities. For instance, groups promoting sobriety for people living with alcohol addiction have been documented since the 1840s in the United States.^3^ Alcoholics Anonymous (1935–) is the best‐known example.^3^ Authors suggest these were spaces to share and validate experiences.^3^ Some groups also offered a model of support as an alternative to medical approaches that tended to stigmatize individuals.^2^, ^8^ For instance, in the late 1960s and 1970s, members of drug user communities mobilized to ensure access to clean injection products to limit the spread of human immunodeficiency virus (HIV) or hepatitis C across the United States and Western Europe.^7^ Some grassroots groups had a strong focus on advocacy. The Alleged Lunatics Friend Society (1845–1863) denounced the abuses in the British madhouse system.^28^ Similar groups formed to protest German involuntary internment laws in the late nineteenth century.^29^ More recently, the Psychiatric Survivors Movement in the 1970s rallied ex‐psychiatric patients across North America to condemn inadequate institutional care and encouraged the involvement of patients in government‐funded mental health services.^30^

Another trend is the formal engagement of community members to deliver healthcare services and support health promotion. Since the 1920s, in rural China, community members have been hired and trained to offer care services to address high rates of preventable illnesses (e.g., infant diarrhoea and dysentery, tetanus, tuberculosis).^5^, ^6^ Between the 1950s and 1970s, similar initiatives emerged worldwide, particularly in low‐ and middle‐income countries, leading to the recognition of their contribution to community participation and health promotion at the Alma‐Ata World Health Organization conference of 1978.^31^, ^32^ In the following years, professional staff shortages and structural adjustment policies imposed by the International Monetary Fund and World Bank limited access to public funding for national programmes, and many collapsed or became smaller in scale.^33^ Those remaining shifted their focus from self‐reliance and health equity to task‐shifting functions and disease‐oriented services.^34^ Involving community members in care also gained popularity since then in high‐income countries, especially to bridge low‐income racial and ethnic minorities to health systems.^33^

Finally, patient navigators are another trend of interest, especially in North America. These programmes focus on removing the barriers disadvantaged patients face when accessing care.^35^ They were documented in Indigenous communities (Aboriginal patients' liaisons) in the 1980s in Canada.^36^ Authors, however, tend to consider the first patient navigation programme as an initiative developed in the 1990s, in Harlem, New York to address low‐income African American women's limited access to breast cancer diagnosis and care.^37^ Navigators were hired from the community to tackle the barriers these women faced when accessing care; for instance, culturally irrelevant information, mistrust of professionals or limited health insurance.^37^ The initial results suggested an increase in early‐stage diagnosis and treatment leading to a rise in survival rates.^37^ This encouraged policymakers to support the deployment of patient navigation across the United States.^37^ Nowadays, some patient navigation programmes hire nurses or social workers from marginalized communities to offer support, rather than community members.^35^ By 2003, patient navigation was integrated in over 200 healthcare programmes in the United States and in other countries like Canada.^35^

## 4 CONCEPTUAL MAP: DISENTANGLING PEERS' ENGAGEMENT IN CARE

**Figure 2:**
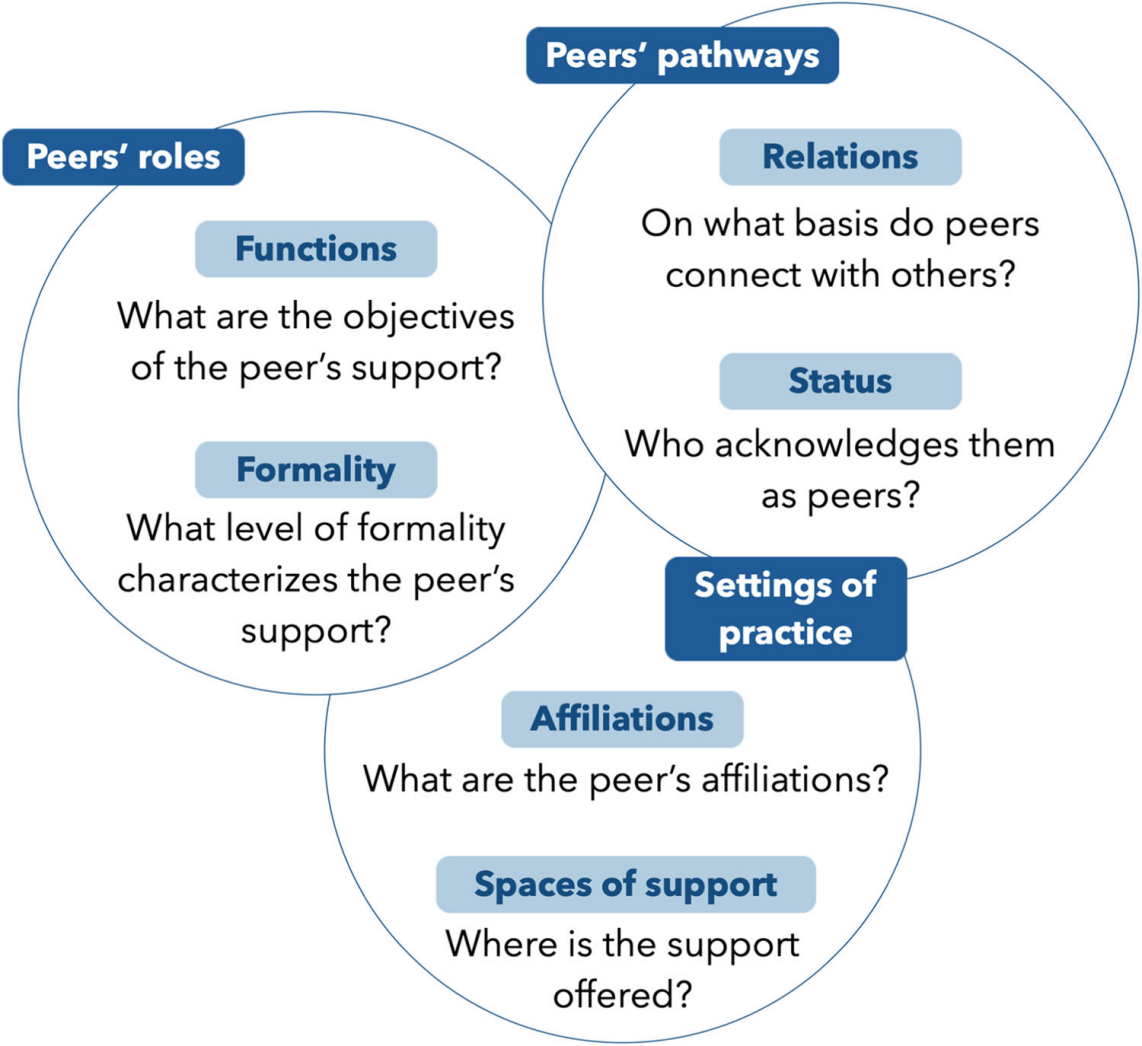
Conceptual map of peers' engagement in care.

The trends discussed above highlight the multifaceted nature of peer support. To disentangle and situate how peers engage in care, we present our conceptual map (Figure 2). The map is built around six key questions focusing on three central facets of peer support: peers' roles, peers' pathways and settings of practice.

## 5 PEERS' ROLES

Peer support can have multiple aims. Peers can seek to improve the self‐management skills of patients, the screening timeline for diseases, reduce social isolation or promote the uptake of treatment. Such objectives may be achieved with formal or informal interventions, based on a few short meetings or frequent and lengthy follow‐ups. To situate peer roles, we propose two questions: first, 'What are the objectives of the peer's support?' and, second, 'What level of formality characterizes the peer's support?' The former reflects on *why* support occurs, whereas the latter focuses on *how* it occurs.

### 5.1 Functions

We delineate five broad types of objectives in peers' support: relationship building, emotional support, navigation, promotion and advocacy. In their own ways, these contribute to accessible, adaptable, tailored and timely care, particularly for marginalized and disadvantaged communities.

*Relationship building* is often the first step in peer support. Peers can seek to establish a connection with a person, to eventually offer practical advice, emotional support or bridge them to services.^2^, ^12^ Peers involved in the knowledge synthesis highlighted how it can take time to build a relationship with individuals, especially those who experience exclusion and prejudice in daily life, workplaces or healthcare institutions. Indeed, mistrust is an important barrier to care for marginalized communities, well documented for instance with people experiencing homelessness, LGBTQ people or undocumented immigrants.^12^, ^13^, ^38^, ^39^ Peers may thus start out to build a relationship by greeting someone when they pass them or having short conversations on weather or leisure, to acknowledge their presence. Building relationships also requires making the time to listen to someone's life story, challenges or concerns, acknowledging they are worthy of being heard and seen.^40^ Offering a space for individuals to be recognized, through and beyond their hardship, peers are able to establish a meaningful connection based on trust.^2^, ^12^ This function of peer support is often fundamental: it paves the way to the other types of support peers can provide.

*Emotional support* is another function of peers' intervention. Emotional support can anchor itself in sharing mutual experiences. Peers can show deep empathy to others, that they understand someone ‘on another level’, as they have experienced these challenges themselves.^12^, ^18^, ^41^ Through discussions, peers can explore ways to walk through hardships, propose other outlooks and shed light on the strengths and skills individuals have to face them.^11^, ^12^, ^42^ Such emotional support implies specific skills, such as authenticity and listening without judgement.^11^, ^41^, ^43^ Systematic reviews have found emotional support contributes to reducing feelings of isolation and fostering a sense of validation.^12^, ^18^, ^41^ By sharing experiences and acknowledging the self‐worth and capacities of individuals, peers act as a model of hope, which can empower them to take action and achieve their own life goals.^2^

*Navigation* is another important role of peers in community care that addresses the complexity of healthcare ecosystems. Navigational support can refer to different tasks, such as referrals and linkages to health and community services, appointment booking, follow‐ups with providers, accompaniments or providing practical assistance for housing, food insecurity, financial aid and even hobbies.^17^ Such interventions require peers to understand the needs individuals might have, and identify the appropriate resources to which to bridge them.^16^ Research suggests that navigational support can facilitate screening of chronic diseases^44^ and help to coordinate care across sectors and providers,^11^, ^45^ which is critical to address the complex social and healthcare needs of disadvantaged groups.^46^

Another function of peers' intervention is the *promotion* of behaviours and knowledge that contribute to health or well‐being. For instance, some peers focus on providing information on the transmission, effect, or treatment of diseases afflicting a community, ranging from tropical diseases to diabetes and HIV.^47^ They can also target at‐risk behaviours or habits, for instance smoking, and conduct health assessment interventions (e.g., blood pressure and weight surveillance).^45^ Some peers distribute medications and harm reduction materials, such as contraceptives or injection kits.^7^, ^48^ These interventions require peers to mobilize medical knowledge about health, disease or well‐being.^49^ However, peers can also draw on experiential knowledge to offer practical advice to cope with hardship^2^ or translate medical information to be culturally relevant and accessible.^47^ Authors found peers can increase engagement in care, fostering self‐efficacy and raising awareness of diseases and treatments.^12^, ^47^, ^50^, ^51^

Finally, peers' interventions frequently have an *advocacy* component or objective. Peers can advocate for the needs of communities, acting as mediators between them and the healthcare system, to render care accessible and adapted.^16^, ^34^, ^52^ Research suggests that peers create collective knowledge and a specific narrative identity that calls into question standardized ways of knowing (e.g., scientific, medical) and current clinical practices.^2^, ^53^ For example, Gurnani and colleagues illustrate how peer educators in India can train police officers to increase their understanding of the realities of sex workers and reduce fear of arrest, which was undermining HIV prevention programmes.^54^ Peers can also contribute to the empowerment of communities, supporting or advocating for their engagement in local initiatives or programmes for instance.^20^, ^30^, ^34^, ^55^

The broad types delineated above highlight how flexible and adaptive peers' interventions can be. Indeed, relationship‐building, emotional support, navigation, promotion and advocacy are not mutually exclusive functions. Peer approaches are multifaceted, building on various emotional, relational and navigational skills, allowing them to tailor support to the needs and goals of individuals and communities.^11^

### 5.2 Formality

Supportive roles can occur in various forms. Peer support relationships can be conceived through a continuum, from informal, spontaneous, mutual aid to more formalized and structured interventions.^21^, ^56^

Peer support occurs ‘naturally’ within existing social networks.^21^ For instance, Worrell and colleagues documented mental health support between friends and partners in LGBTQ+ communities. They provided individuals a space to be listened to or ask for advice during a crisis.^39^ Research suggests such support also occurs within other marginalized communities, for exemple between friends and partners living with HIV.^56^ This support may, however, put peers at risk of distress, or make it difficult to assert limits on the support offered.^39^ Informal and spontaneous support has also been documented among individuals regrouped by life circumstances, for instance psychiatric hospitalization or homelessness.^57^, ^58^ Organizations or programmes might choose to capitalize on such networks by creating and sustaining opportunities for mutual support.^56^ Informal networks hold the potential to offer or receive support directly, in ‘day‐to‐day living environments’.^56^

At the other end of the continuum, support occurs through ‘created’ or 'formalized' relationships.^21^, ^56^ For instance, some peers intentionally provide support, as a part of a function within a programme, and may have received training for it.^59^ The framework of intervention might still be more or less structured, open to spontaneity or the client's emerging needs. For instance, some mental health peer support workers tailor their support by making the time to listen to a client's needs and their desire to engage in care.^60^ Other peers have predefined goals to achieve and follow protocols.^61^ In certain community health worker programmes, peers have specific tasks to accomplish, like evaluating and administering malaria treatment based on a fever management algorithm.^34^ Formally fostered relationships may come with the assumption that the peer providing support is ‘more advanced’ in their recovery, or a ‘model’ to follow, as illustrated by terms such as ‘peer mentor’ or ‘peer leader’.^12^, ^61^ Some authors caution that formalization of peer support might create interpersonal distance that, when too great, questions the authenticity of peer relations and recreates power relations between peers.^18^, ^59^

While it may be useful to reflect on the format of peer support that is better suited to a project or initiative, research suggests peers hold similar roles regardless of the formality of their interventions. Whether in existing or created social networks, peers can provide a space to talk about personal issues, actively listen and encourage certain behaviours.^56^, ^58^ Individual preferences and contextual factors may play an important part in what support people seek, and from whom; some might be more comfortable discussing with close friends, while others prefer the anonymity of formal support.^56^

## 6 PEERS' PATHWAYS

The discussion above hints at a key feature of peer interventions: they anchor themselves in life courses and social identities. Peer support implies a mutual recognition process, through which individuals see each other as peers, ‘one of’ a group or community.^2^, ^62^ The pathways to becoming a peer differ from those of healthcare providers, managers or policymakers, although with their increased engagement in formal healthcare, related mechanisms now recognize peer status (e.g., training programmes). To situate models of peer support, understanding peers' pathways is fundamental, especially since it is linked to what enables peers to establish relations and rapport with others. To do so, we propose two questions: first, ‘On what basis do peers connect with others?’ and, second, ‘Who acknowledges them as peers?’

### 6.1 Peers' relations

Some peers relate with others based on ‘lived experience’. Authors argue that by experiencing health and/or social challenges, peers acquire unique knowledge and skills, which they mobilize to support others.^2^ Chronic disease, homelessness, addictions, mental illness, loss and grief are all examples of lived experiences leading peers to engage in care. According to different studies, the intimate or ‘insider’ knowledge gained through hardship helps peers to build relationships and serves as a foundation for trust and mutual understanding.^2^, ^12^, ^42^ Sharing the experience of health and/or social challenges can normalize and validate these experiences, reducing feelings of stigma or loneliness that they may come with.^18^, ^42^

Some peers also rely on their social identities to establish rapport. For example, in North America, Rankin and colleagues^13^ report that being an Indigenous patient navigator helps to establish trust and relationships with Indigenous people. This is critical considering that healthcare systems still tend to reproduce colonial practices, and often rely on Eurocentric conceptions of health and care.^13^ In their study of a community health worker programme in Surabaya, Indonesia targeting perinatal depression, Surjaningrum and colleagues^43^ mentioned similar findings about the peer's gender. Participants stated they preferred working with women, reflecting that women were more likely to “promote comfort and less shameful feelings”.^43^ Sharing identities might thus help peers to connect, as it presupposes the possibility of having experienced similar situations or, at least, of being understood from an ‘insider’ standpoint.

Another way peers connect is through their membership and engagement in a local or geographic community. Some programmes recruit local members to educate and provide advice on health and illness; for instance, in Roma communities in Europe or Latino neighbourhoods in the United States.^15^, ^63^ While these peers also share certain identities (e.g., ethnicity or language) with those they support, what distinguishes them is that they are embedded in the local community served.^19^, ^20^ As such, they closely understand the challenges the community faces when accessing local services and their actual needs or concerns.^15^, ^64^ As community members, they can translate services and information to be culturally appropriate, and advocate for their community's needs to local healthcare representatives or providers.^19^, ^20^ In low‐ and middle‐income countries, some communities select the peers to ensure they are trusted local leaders, and to promote community participation in the programme's design.^20^

Certain peers mobilize lived experiences, social identities, or their engagement in a geographic community to build relationships. They may even draw simultaneously on each of these bases for peer relations to connect with others. Local communities are bound by social identities and lived experiences modulated by them. In daily life, these distinctions are permeable. Hence, using such categories to sort out how peers establish rapport when situating a programme or an initiative may be more useful from a theoretical perspective, as opposed to an empirical one.

### 6.2 Peers' status

Deeply linked to the way peers connect with the people they accompany is the question of who acknowledges their peer status. The examples discussed above illustrate that one is foremost seen as a peer by the people they support, through mutual recognition processes at play during interventions.^2^, ^62^ This may limit the possibility of planning or predicting the way peers will ‘in fact’ connect. In our discussion with practising peers in Canada, they highlighted that sharing a characteristic or an experience does not systematically enable or facilitate relationships. Other ‘ingredients’ may be necessary; for instance, having a sense that the peers' presence is authentic, genuine and safe. This points to the possibility of peer status being recognized through an attitude or interpersonal skills.

Peer status may also be acknowledged by an organization or institution. Increasingly, peers offer support within formal jobs or functions in programmes led by community organizations or healthcare institutions.^2^, ^9^ In these contexts, peers are solicited for their unique expertise, perspective or knowledge, within the organization. Their roles and functions as a peer may be formally delineated according to the programmes' objectives, and peers might even have an employee card or access to an office space.^40^ Organizational or institutional recognition of peer status can also take the form of receiving mentorship or support from healthcare providers or representatives.^20^ While such formal recognition can benefit peers' interventions, enhancing their credibility in the community served,^65^ they also render them accountable to the organizations' or institutions' goals, sometimes contrasting those of individuals or communities.^66^

Peer status is also acknowledged increasingly through training and certification programmes.^9^, ^55^ These have important implications for peers, as they can influence their skills and competencies, and the way they will mobilize their experiential knowledge and/or identities. For instance, training can aim to foster confidentiality practices and explore different ways to approach communication.^12^, ^20^ It might also target ways peers could manage relationship boundaries; for instance, when they know and live in the community they serve.^38^, ^67^ Beyond training, research suggests that certification programmes might contribute to peers' compensation (e.g., wages, salary and incentives) and their official recognition as a workforce in healthcare ecosystems.^52^, ^55^, ^63^ Some authors, however, call for caution. Training programmes can reproduce social inequalities (e.g., requiring peers to have a diploma beforehand),^68^ or formalize peers' approach, which could impede their ability to build trust and reciprocal relationships.^55^, ^63^

While training programmes or institutions can acknowledge and recognize one's status as a peer, research suggests it is foremost certain personal qualities and attributes, fostered across the life course, that enable peers to take on supportive roles.^2^, ^69^ Accounting for peers' experiences with workplaces, training or institutions remains of importance, especially with the increased integration of peers in formal healthcare structures.^2^, ^9^ The discussion above highlights the intersecting and multiple experiences that might inform their current interventions, and enable them to hold supportive roles across different settings.

## 7 SETTINGS OF PRACTICE

Reflecting on the settings of peers' interventions is complex. Peers might work for a community organization, a hospital‐based programme or an independent self‐help group,^55^ but conduct the majority of their interventions in other spaces; for instance, in the streets, a home or institutions (e.g., justice courts, prisons). *Who* peers work for and *where* they offer support are two intricate matters. Both shape their roles and create opportunities or challenges in their practice. To conclude our conceptual map, we propose two final questions: first, ‘What are the peer's affiliations?’ and second, ‘Where is the support offered?’ To reflect on them, we build the discussion on the community‐clinical continuum.

### 7.1 Peers' affiliations

Some peers offer support independently, without any formal association with a programme, organization or institution. They are affiliated foremost with a community or a group. Mutual aid and informal support groups constitute significant examples, where peers work ‘outside’ formal healthcare systems (see Section 3). In her study of an alternative self‐help group for ‘psychiatric survivors’, Laws^70^ shows peers can actively seek the separation from clinical space to generate counter‐narratives to medical conceptions of recovery. Offering support independently might be a response to the stigmatization and harm experienced by communities from formal healthcare services.^39^ Authors suggest the recognition of shared exclusion and harm plays a role in such mutual aid.^71^ Offering support independently from organizations or institutions, however, can be difficult, limiting access to support and resources for the peers themselves.^39^

Alternatively, numerous peers work for community‐based organizations, which may or not be peer‐run.* Some authors suggest that, compared to clinical settings, these tend to operate with less hierarchical governance and hire individuals with diverse backgrounds, including paraprofessionals.^55^, ^72^ According to Jones and colleagues, peer‐run organizations in mental health offer peers greater representative power, more influence on governance or service delivery, and opportunities for management positions.^73^ While community‐based or peer‐run organizations are not immune to power relations, research suggests that less hierarchical workplace cultures help to protect peers' distinctive role and provide a more flexible framework for interventions.^14^, ^72^

Finally, peers can also work for clinically based organizations. Authors suggest that peers in these settings tend to focus on offering direct services to clients, rather than holding management positions.^55^, ^73^ Working for clinically based organizations can encourage peers to adapt their interventions and language to fit medical knowledge, which is often *the* legitimate form of knowledge in clinical settings.^18^ This might limit their ability to use lived experiences to question instituted practices,^73^ or render them accountable to healthcare organization needs and objectives, rather than communities.^47^ On the other hand, such affiliations can facilitate access to healthcare providers,^73^ and in some cases better working hours or workloads.^14^ In low‐ and middle‐income countries, research also highlights that linkages to formal healthcare systems contribute to peers' credibility when working in their community.^48^, ^65^

Peer affiliations may create opportunities and challenges for their work, and so do the spaces in which they conduct their interventions. For instance, a peer might work *for* a clinic and be asked, during an intervention or as part of a formal agreement, to visit a community organization, thus interacting with its actors and culture.

### 7.2 Peers' spaces

Peers are called to offer support in different spaces of daily life: in homes, churches, in the street, cafes, restaurants and so on.^2^, ^9^, ^21^, ^70^ This can happen informally, between friends and family, but also with peers hired to reach individuals in these spaces. Outreach work in the streets, for instance, has been an important part of peer support with people living with substance use or experiencing homelessness, reaching them ‘where they are’.^7^, ^62^ Experiential or ‘insider’ knowledge allows peers to know where to go to meet and connect with such marginalized groups, whom healthcare systems still struggle to reach.^7^ Another example of peers working in community settings peer programmes in isolated communities, who may see individuals they support outside their working hours, challenging boundaries between their peer role and their community embeddedness.^67^ Offering support directly in communities may facilitate peers' ability to bridge disadvantaged individuals to healthcare services.^7^, ^11^ As such, explicit referral mechanisms or ongoing collaborative relationships with local services may facilitate their interventions.^20^, ^48^, ^65^

Such relationships may even be beneficial when peers offer support in organizational and institutional settings. Peers can, for instance, introduce someone to community organizations, such as food banks or shelters, going physically with them to break the associated stigma.^40^ Peers involved in the knowledge synthesis, working with disadvantaged communities, especially Indigenous people, reflected that they accompanied clients to clinics or hospitals to ensure their rights were respected. However, in institutional settings, peers can also face prejudice, particularly when their peer status is anchored in marginalizing experiences.^55^, ^74^, ^75^ In community organizations or healthcare institutions, research suggests that team members' understanding and recognition of peers' role plays an important part in interprofessional collaborations.^59^, ^74^ In an acute inpatient psychiatric hospital in London, Galloway and Pistrang found that some staff  discouraged or closely monitored informal support among patients due to perceptions of potential harm and risks.^58^ This example highlights that, wherever peers offer support, in institutions or communities, they interact with various actors of health ecosystems (e.g., friends, citizens, community workers, providers and managers), whose understanding of their role is crucial to successful collaboration.

## 8 DISCUSSION

In this paper, we presented a conceptual map of peers' engagement in care, based on a 3‐year knowledge synthesis. The project involved peers, providers and researchers in Canada, and international literature on the subject. We proposed six ‘key questions’ to situate initiatives or programmes according to the peer's roles, pathways and settings of practice. Each question acts as a tool to disentangle the heterogeneous body of approaches, goals and models of engaging with peers in care.

Throughout the knowledge synthesis, peers and providers reflected on how peer support programmes tend to operate in silos. These were segmented across the communities targeted (e.g., migrants, Indigenous people, women), the diseases or issues addressed (homelessness, mental illness, substance use), the peer's main role (navigation, support, outreach) and their setting of practice (community organizations, hospitals, clinics), limiting their ability to exchange knowledge with other initiatives or programmes. Our exploration of international literature suggests research is also organized around these silos. This prompted us to develop a conceptual map that would help to navigate the specificities of, and possible connections between, the diverse models—or ‘silos’—of peers' engagement in health and community care.

The conceptual map is an introductory and exploratory step. Our approach to scientific and grey literature was flexible, iterative and outward‐looking, rather than systematic and inward‐looking. This enabled us to search and connect knowledge and literature that are related but rarely brought together and connected. Our approach also allowed us to take into account the knowledge of the peers involved in the conceptualization of the map and investigate their priorities more closely. Showing the breadth, depth and richness of peers' engagement in care, this synthesis does not pretend to offer an exhaustive overview of the field. While analytical questions and conceptualizations are useful tools, they might only be reductive when compared to the multifaceted and compounded nature of peer support.

Future research could investigate more deeply the similarities and differences between models of peer support, particularly since existing knowledge suggests that peers' engagement in care holds great potential to integrate health and community sectors. Peer support helps to bridge underserved communities to healthcare, cutting across health, community and institutional sectors, services and providers.^2^, ^11^, ^16^, ^17^ Peers achieve this impact through their ability to connect, establish trust and build relationships and to shed light on individuals' strengths and skills, empowering them to engage in care.^2^, ^12^, ^18^ Peers also advocate for the actual needs of these groups, highlighting barriers to care and supporting the adaptation of services to be relevant and safe.^16^, ^19^, ^20^, ^52^ Peer support is a way for healthcare systems to engage with equity‐deserving groups,^38^, ^55^, ^59^ which is critical to tackle the compounded and complex barriers they face in care.^46^ Partnering with peers contributes to accessible, coordinated and adaptive care across sectors, providers and communities.

These outcomes are not, however, straightforward: they require different structures or mechanisms to be actualized. Research on the implementation and integration of peers across healthcare ecosystems has identified multiple ways peers can be supported to conduct their work, ranging from access to salaries, compensation and material resources (such as laptops, phones and bus passes) to emotional support after triggering events.^10^, ^40^, ^65^, ^72^ Partnering with peers is neither a linear process nor a simple one. It relies on the commitment and collaboration of different actors who possess different, sometimes conflicting, knowledge, values and skills: researchers, managers, providers, policymakers, health representatives, citizens, peers and communities. Useful guidelines and frameworks exist to support such collaborations and engage meaningfully and ethically with peers in care.^76^, ^77^, ^78^ To work together and exchange, we believe these actors must also build on a common language, to recognize each other, establish dialogue and access existing knowledge beyond labels and silos. Our conceptual map offers questions to build such connections and navigate the multifaceted nature of peer engagement in care.

## 9 CONCLUSION

Peer support is multifaceted. The wide range of labels and models may be daunting at first, but highlights the versatility of peer interventions in healthcare ecosystems, internationally. This article is a small step towards laying the foundation for a common language to bridge initiatives and support knowledge exchanges between peers, researchers, providers and policymakers that work in parallel, but deeply related, worlds of peer support. Further research could explore bridges that can strengthen our ability to learn from each other across initiatives and contexts.

## AUTHOR CONTRIBUTIONS

**Andreea‐Cătălina Panaite**: Conceptualization; methodology; investigation; writing— original draft; writing—review and editing; visualization; project administration. **Odile‐Anne Desroches**: Conceptualization; investigation; writing—original draft; writing —review and editing; visualization. **Émilie Warren**: Conceptualization; methodology; investigation; writing—review and editing; writing—original draft. **Ghislaine Rouly**: Conceptualization; writing—original draft; writing—review and editing; resources; supervision. **Geneviève Castonguay**: Project administration; writing—original draft; writing—review and editing; conceptualization. **Antoine Boivin**: Resources; writing—review and editing; writing—original draft; funding acquisition; investigation; conceptualization; methodology; supervision.

## CONFLICT OF INTEREST STATEMENT

The authors declare no conflict of interest.

## Data Availability

The new data generated are not publicly available due to privacy or ethical restrictions. The authors declare the absence of shared data.

## Footnotes

* Community or peer‐run organizations may be linked to health agencies through financial support or partnerships.
